# miR-429 inhibits cells growth and invasion and regulates EMT-related marker genes by targeting Onecut2 in colorectal carcinoma

**DOI:** 10.1007/s11010-013-1950-x

**Published:** 2014-01-10

**Authors:** Yingnan Sun, Shourong Shen, Xiaoping Liu, Hailin Tang, Zeyou Wang, Zhibin Yu, Xiayu Li, Minghua Wu

**Affiliations:** 1 Hunan Key Laboratory of Nonresolving Inflammation and Cancer, Changsha, Hunan People’s Republic of China; 2 Department of Gastroenterology, Third Xiangya Hospital, Central South University, Changsha, 410013 Hunan People’s Republic of China; 3 Cancer Research Institute; Disease Genome Research Center; Key Laboratory of Carcinogenesis and Cancer Invasion, Ministry of Education; Key Laboratory of Carcinogenesis, Ministry of Health, Central South University, Changsha, Hunan People’s Republic of China; 4 Sun Yat-Sen University Cancer Center, State Key Laboratory of Oncology in South China, Collaborative Innovation Center for Cancer Medicine, Guangzhou, Guangdong People’s Republic of China

**Keywords:** miRNA, miR-429, Colorectal carcinoma, TGF-β1, Snail, ZEB2

## Abstract

The 5-year survival rate for colorectal cancer is approximately 55 % because of its invasion and metastasis. The epithelial–mesenchymal transition (EMT) is one of the well-defined processes during the invasion and distant metastasis of primary epithelial tumors. miR-429, a member of the miR-200 family of microRNAs, was previously shown to inhibit the expression of transcriptional repressors ZEB1/delta EF1 and SIP1/ZEB2, and regulate EMT. In this study, we showed that miR-429 was significantly downregulated in colorectal carcinoma (CRC) tissues and cell lines. We found that miR-429 inhibited the proliferation and growth of CRC cells in vitro and in vivo, suggesting that miR-429 could play a role in CRC tumorigenesis. We also showed that downregulation of miR-429 may contribute to carcinogenesis and the initiation of EMT of CRC by targeting Onecut2. Further researches indicated that miR-429 inhibited the cells migration and invasion and reversed TGF-β-induced EMT changes in SW620 and SW480 cells. miR-429 could reverse the change of EMT-related markers genes induced by TGF-β1, such as E-cadherin, CTNNA1, CTNNB1, TFN, CD44, MMP2, Vimentin, Slug, Snail, and ZEB2 by targeting Onecut2. Taken together, our data showed that transcript factor Onecut2 is involved in the EMT, migration and invasion of CRC cells; miR-429 inhibits the initiation of EMT and regulated expression of EMT-related markers by targeting Onecut2; and miR-429 or Onecut2 is the important therapy target for CRC.

**Electronic supplementary material:**

The online version of this article (doi:10.1007/s11010-013-1950-x) contains supplementary material, which is available to authorized users.

## Introduction

Colorectal cancer (CRC) is one of the most common gastrointestinal malignant tumors in the world and the third leading cause of cancer death worldwide [1]. There are about 1.2 million new-onset patients around the world each year, and about half of them die within 5 years, and invasion and metastasis is the leading cause of death of patients with CRC [2]. Tumor invasion and metastasis is a complicated process involving in various factors and multiple steps. One of the well-defined processes that occurred during the invasion and distant metastasis of primary epithelial tumors is called the epithelial–mesenchymal transition (EMT) which was commonly observed in various types of malignant tumors including CRC [3, 4]. A number of studies have indicated that growth factors, such as transforming growth factor-β (TGF-β), epithelial growth factor (EGF), hepatocyte growth factor (HGF), insulin-like growth factor (IGF), vascular endothelial growth factor (VEGF), and platelet-derived growth factor (PDGF) are the activators of EMT [5, 6].

TGF-β/Smad signaling pathway; mitogen-activated protein kinase (the mitogen-activated protein kinase MAPK) signaling pathway; Src kinase signaling pathway; the Rho kinase pathway; phosphatidylinositol-3 kinase (phosphatidylinositol 3 kinase, PI3K) signaling pathway; Jagged1/Notch signaling pathway; the Wnt/β-catenin signaling pathway; and other cell proliferation, apoptosis, metastasis-related signaling pathways have been involved in the initiation of EMT, and these pathways have the rich cross-talk in the EMT process [7–10]. Studies have confirmed that many transcription factors including Twist, Snail, Slug, ZEB1, ZEB2, E27, FOXC2, Goosecoid, and NF-κB are the activators of EMT, and play an important role in tumor metastasis [11–16]. Recent studies have identified microRNAs as key players in EMT-associated cancer metastasis. For example, miR-1 and miR-200 inhibit EMT and tumorigenesis via Slug-independent mechanisms [17]. miR203/Snail-1 and miR200/ZEB construct two fundamental negative feedback loops of EMT core network [18]. The members of the miRNA-200 family (miR-141; miR-200a, b, c; and miR-429) and miR-205 play important roles in the EMT-associated cancer metastasis [19–22]. ZEB1 and ZEB2 are known targets of miRNA-200 family and inhibition of these regulating microRNAs has been shown to be sufficient to induce EMT in a variety of cell types [23].

On the basis of miRNA–RNA network of CRC constructed by our previous research, we found that miR-429, miR-18, and miR-490-3p are the core of the target genes network [24]. miR-429, a member of the miR-200 family of microRNAs, was previously shown to inhibit the expression of transcriptional repressors ZEB1/delta EF1 and SIP1/ZEB2 and regulate EMT, which represents an important early step during metastasis [25]. We, thus, proposed that the deregulation of miR-429 would influence the pathological progress of CRC. Validation of miR-429 with expression in extended clinical sample and cell biology functional assay proved our hypothesis that miR-429 would act as an anti-EMT and invasion miRNA.

## Materials and methods

### Patients and tissue samples

The patient material including primary tumor (II and III stages) and the corresponding adjacent normal tissue (paracancerous tissues) were collected at the Xiangya Affiliated Hospital, Xiangya Second and Third Affiliated Hospital, Central South University between the years 2009 and 2011. Written informed consent was obtained from all the study participants. Collections and using of tissue samples were approved by the ethical review committees of the Xiangya Hospital Ethic Committee of Central South University.

### Cell lines and cell culture

Human colorectal carcinoma cell lines SW-480, SW-620, and HT-29 were obtained from the Cell Bank of Shanghai (China) and were cultured in RPMI 1640 medium supplemented with 10 % fetal calf serum, 100 U/ml penicillin, and 100 lg/ml streptomycin at 37 in a 5 % CO_2_ incubator. TGF-β1-stimulation experiments were performed with recombinant human TGF-β1 (10 ng/ml; RD systems).

### Real-time quantitative PCR analysis

The total RNAs were extracted from cells with TRIZOL reagent (Invitrogen, Wuhan, China). RT reactions were performed by means of SYBR-green-containing PCR kit (GenePharma, Shanghai, China).The primers for real-time PCR to detect miR-429 (Forward primer: 5′-UAAUACUGUCUGGUAAAACCGU-3′; Reverse primer: 5′-UUCUCCGAACGUGUCACGUTT-3′) were designed based on the miRNA sequences provided by the Sanger Center miRNA Registry and were synthesized and purified by Shanghai Gene-Pharma biotechnology limited company (Shanghai, China). The primers of EMT-related marker genes were synthesized and purified by Shanghai Shengong Biotechnology Limited Company (Shanghai, China). All real-time PCR were performed on the BIO-RAD IQTM5 Multicolor Real-Time PCR detection System (Bio-Rad, USA).

**Table:** The primers of EMT-related marker genes

Gene names	Forward primer (5′–3′)
	Reverse primer (5′–3′)
GAPDH	f: 5′-CGACCACTTTGTCAAGCTCA-3′
	r: 5′-AGGGGTCTACATGGCAACTG-3′
E-cadherin	f: 5′-TTCTGGAAGGAATGGAGGAGTC-3′
	r: 5′-ACCTGGAATTGGGCAAATGTG-3′
Vimentin	f: 5′-AGATGGCCCTTGACATTGAG-3′
	r: 5′-TGGAAGAGGCAGAGAAATCC-3′
CTTA1	f: 5′-GGGGATAAAATTGCGAAGGAGA-3′
	r: 5′-GTTGCCTCGCTTCACAGAAGA-3′
CTTB1	f: 5′-TTGTTGTGTTACAATGCTGCCT-3′
	r: 5′-GTGCTTTTTGAGCTAGATCCCA-3′
TFN1	f: 5′-GGTGACACTTATGAGCGTCCTAAA-3′
	r: 5′-AACATGTAACCACCAGTCTCATGTG-3′
CD44	f: 5′-AATCCCTGCTACCAATATGGACT-3′
	r: 5′-TCCACCTGTGACATCATTCCTAT-3′
MMP2	f: 5′-CAACTACGATGATGACCGCAA-3′
	r: 5′-GTGTAAATGGGTGCCATCAGG-3′
ZEB1	f: 5′-GCACAACCAAGTGCAGAAGA-3′
	r: 5′-GCCTGGTTCAGGAGAAGATG-3′
SNAILl	f: 5′-GCTGCAGGACTCTAATCCAGAGTT-3′
	r: 5′-GACAGAGTCCCAGATGAGCATTG-3′
Slug	f: 5′-AGATGCATATTCGGACCCAC-3′
	r: 5′-CCTCATGTTTGTGCAGGAGA-3′
Onecut2	f: 5′-CATACTCAAGCGGGACCTTCC-3′
	r: 5′-TTGGTGGAACTGGGAGTCTAA-3′

### In situ hybridization and immunochemistry analysis

miR-429 miRCURYTM LNA custom detection probe (Exiqon, Vedbaek, Denmark) was used for in situ hybridization (ISH). The sequence 5′–3′ (enhanced with LNA) was acggttttaccagacagtatta with DIG at the 5′ and 3′ ends. Hybridization, washing, and scanning were carried out according to the manuals and protocols provided by the Exiqon life science department. Immunohistochemistry (IHC) studies were performed using the standard streptavidin/peroxidase staining method as described previously. Image analysis and total gray value were estimated by the GSM-2000P pathology image analysis system [26].

### MTT assays

Logarithmically growing SW-480, SW-620, and HT-29 cells were seeded in 96-well plates (5 × 10^3^ cells/100 μl medium/well) for 6 h. The culture medium was replaced after 24 h with fresh medium containing different concentrations (2.5, 5.0, and 7.5 ρM) of miR-429 mimics. Cells were incubated in the presence of the mimics for 24, 36, 48, 60, and 72 h and then incubated for 4 h in the presence of 20 μl MTT solution (5 g/l, Sigma, Beijing, China). DMSO was added (100 μl/well) and OD values at 490 nm were recorded with the ELX 800 absorbance Microplate Reader (BioTak, Winooski, VT), scramble was used as the control. The assay was performed three times with eight replicates.

### Luciferase assays

The 3′-untranslated regions (UTRs) of the Onecut2 gene were synthesized and annealed, then inserted into the pMIR-REPORT(TM) Luciferase vector (Ambion), using the Hind III (aagctt) and Spe I (actagt) site downstream from the stop codon of luciferase (Luc-wt). We also generated an insert with deletions of 4 bp from the site of perfect complementarity of the Onecut2 gene (Luc-mut).

pMIR-Onecut2-wt:

Forward primer: 5′-CTAGTTATTATGGGTACTTTAAA GTCAGTATTT A TCAAG AAAGGGAACTTGAA-3′.

Reverse primer: 5′-AGCTTTCAAGTTCCCTTTCTTGATAAATACTGACTTTAAAGTACCCATAATAA-3′.

pMIR-Onecut2-mut:

Forward primer: 5′-CTAGTTATTATGGGTACTTTAAAGTTATCAAGAAAGGGAACTTGAA-3′.

Reverse primer: 5′-AGCTTTCAAGTTCCCTTTCTTGATAACTTTAAAGTACCCATAATAA-3′.

Luc-wt or Luc-mut was cotransfected with miR-429 mimics into CRC cells. The pMIR-REPORT™–β galactosidase control vector was transfected as a control. Luciferase activity was measured in cell lysates 48 h after transfection using the Dual-Light luminescent reporter gene assay kit (Applied Biosystems). The pMIR-REPORT™–β-gal control vector was cotransfected as an internal control to correct the differences in both transfection and harvest efficiencies. Transfections were done in duplicates and repeated at least thrice in independent experiments.

### Western blot

Western blot was performed as previously described [4]. Rabbit polyclonal primary antibody against Onecut2 and mouse monoclonal antibody GAPDH was purchased from Cell Signaling Technology (Beverly, MA, USA) and Santa Cruz Biotechnology Inc., (Santa Cruz, CA).

### Construction of pcDNA3.1–Onecut2 plasmids

Briefly, the full length of Onecut2 gene which was amplified by PCR was linked into pcDNA3.1 vector. The primer was designed by primer 3 software. Forward primer: 5′-CACTTGGCAGACCTCTCCTC-3′; Reverse primer: 5′-ACTGTATGGAGGCCCA GTTGTCT-3′

### Wound-healing assay

CRC cells seeded in 6-well plates to be long to near saturation and were “wounded” by removing a line of cells with disinfected Eppendorf Tip. After washing with FBS-free medium, the wound areas were photographed under a microscope. The cells were treated in triplicate with 20 ng/ml TGF-β in FBS-starved medium. Their migrations were monitored at 24- and 48-h post-treatment.

### Matrigel chamber invasion assay

The diluted matrigel (BD Biosciences) was added to the upper well of the Transwell chamber (Corning Inc., Corning, NY), and reconstituted for 1 h at 37 °C. The cells were starved overnight in serum-free medium and re-suspended at a concentration of 2.5 × 10^5^ cells/ml in serum-free medium containing 0.1 % bovine serum albumin. 0.2 ml cell suspension was added to the top of each well, and a 10 mg/ml fibronectin solution was added to the bottom well of the chamber as a chemoattractant. After 36 h, the cells that had not invaded were removed from the upper surface of the filters using a cotton swab. The cells that had invaded to the lower surface of the filter were fixed with methanol, stained with H&E, and 5 random fields (40×) were counted. The data were expressed as the mean value of cells per field in triplicate in two independent experiments.

### Tumor formation assay in nude mice

Cells were washed once with PBS and subcutaneously injected into the flank region at a concentration of 2 × 10^7^ cells/mouse (5-week-old male nude mice). 10 mice were randomly divided into two groups (*n* = 5). Aliquots of 40 μl of PBS containing 1 μg of mature miR-429 or scramble were directly injected into the tumor. At the end of each study, animals were sacrificed and tumors were collected and divided for either storage in RNA later (TaKaRa Inc., Dalian, China) following the manufacturer’s instructions for the following RNA extraction, or fixed in formalin for ISH and IHC. Image analysis and total gray value were estimated by the GSM-2000P pathology image analysis system (Heima Zhuhai, China). Tumor growth was monitored by caliper measurement once or twice a week for at least 5 weeks. Tumor volume was calculated as follows: *V* = *L* × *l* ^2^ × 0.5, where *L* and *l* represent the larger and the smaller tumor diameters, respectively. The animal experiments were performed in accordance with the institutional guidelines.

### Statistical analysis

Differences between groups were tested by a Student’s *t* test or one-way analysis of variance (ANOVA) using the SPSS 13.0 program (SPSS, Inc., Chicago, IL, USA). Spearman’s correlation test was used to evaluate the pairwise expression correlation between miR-429 and Onecut2 in CRC.

## Results

### miR-429 inhibits proliferation and tumorigenesis in CRC cell

**Fig. 1:**
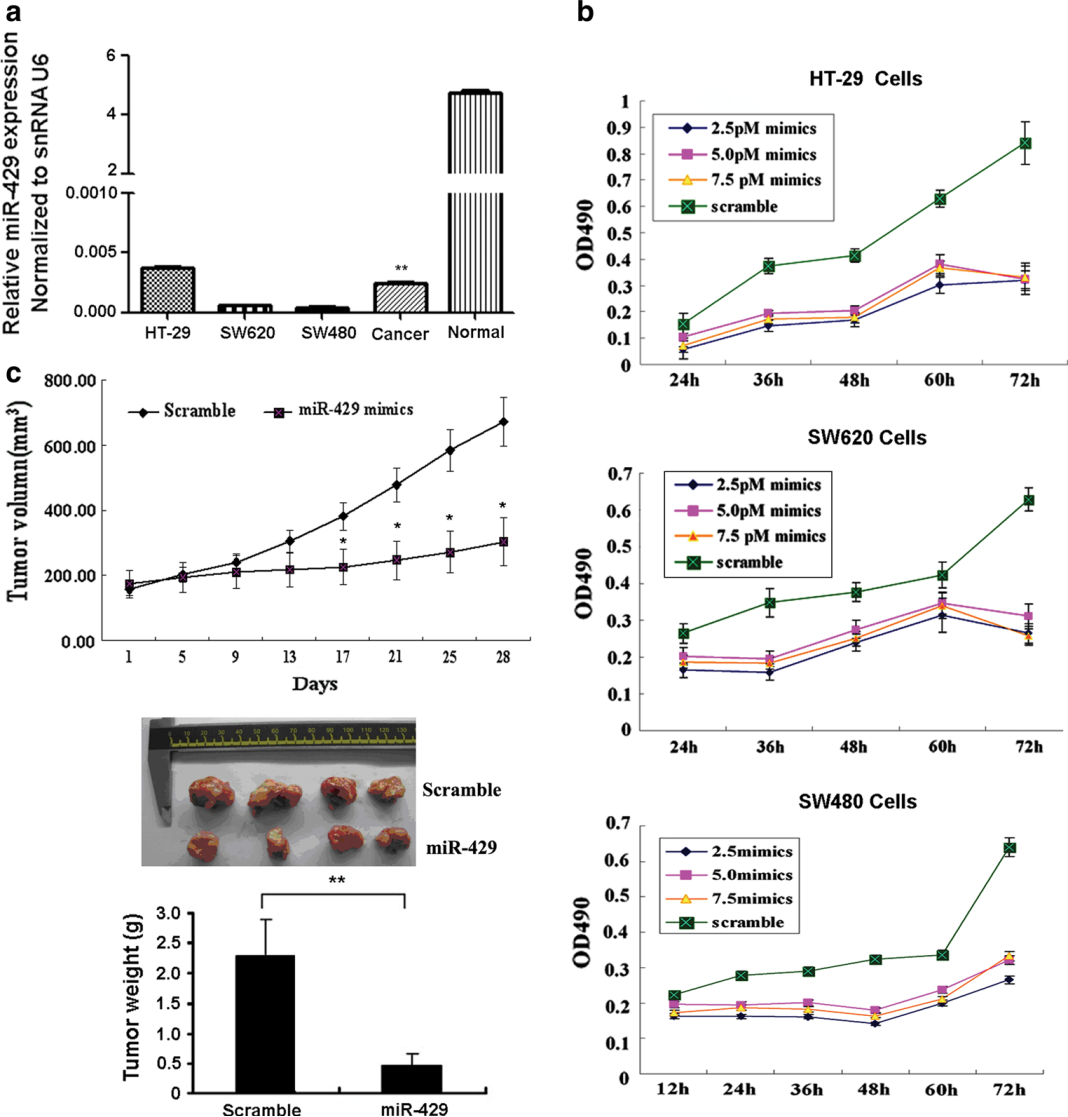
miR-429 inhibits proliferation and tumorigenesis in CRC cells. **a** miR-429 downregulated expression in the CRC tissues (10 cases II and III stage CRC tissues and 10 cases corresponding to the adjacent normal tissue) and multiple cell lines (HT-29, SW620, and SW480 cell). **P < 0.01 compared to the expression of CRC tissues. **b** miR-429 inhibited the cell proliferation of CRC cells for different times in vitro. **c** miR-429 attenuated CRC growth in mouse xenograft models when aliquots of 40 μl of PBS containing 1 μg of mature miR-429 or scramble were directly injected into the tumor. *P < 0.05; **P < 0.01

In order to evaluate the anti-proliferation and anti-tumorigenesis of miR-429, in vitro and in vivo experiments were carried out. The expression of miR-429 was validated by real-time PCR in both CRC tissues and cell lines. miR-429 is significantly downregulated in both II and III stage CRC tissues and cell lines compared to that of the corresponding adjacent normal colon mucosa (*P* < 0.01) (Fig. 1a). The anti-proliferative effect of miR-429 was evaluated in CRC cell lines including SW480, SW620, and HT-29 which were transfected with miR-429 mimics and scramble control in dose (2.5, 5.0, and 7.5 pm) and time (24, 36, 48, 60, and 72 h). The transfection of miR-429 is successful (Fig. 1a). Although MTT measurement did not show a significant dosage effect (*P* > 0.05) (Fig. 1b), it is obvious to notice the significant anti-proliferative effect of miR-429 mimics especially at 72 h of transfection comparing to the scramble-treated cells (Fig. 1b). Thus, we selected the least concentrations of 2.5 pm and 72 h as the standard transfection protocol in the further studies.

Encouraged by the results in vitro, we took a further step toward the assessment of the feasibility of the direct in vivo miR-429 treatment. Highly metastatic potential CRC cell line SW620 was subcutaneously injected into both the flanks of nude mice in order to yield tumors that were then treated by direct intratumoral injection as soon as they became clearly palpable. For each mouse, the tumor on one flank was injected with miR-429, while the control lateral tumor was injected with a control scramble. The growth curves of miR-429-treated versus scramble-treated tumors are compared in Fig. 1c. As shown, the two curves slowly become divergent until they reach statistically different values at 17 days after the first day of injection (Student’s *t* test, *P* < 0.05). In accordance with this observation, the average weight of the treated tumors at the end of the experiment was significantly reduced as compared to that of the control tumors (Student’s *t* test, *P* < 0.01).

### miR-429 targets ONECUT2 and decreases its expression in CRC cells and tissues

**Fig. 2:**
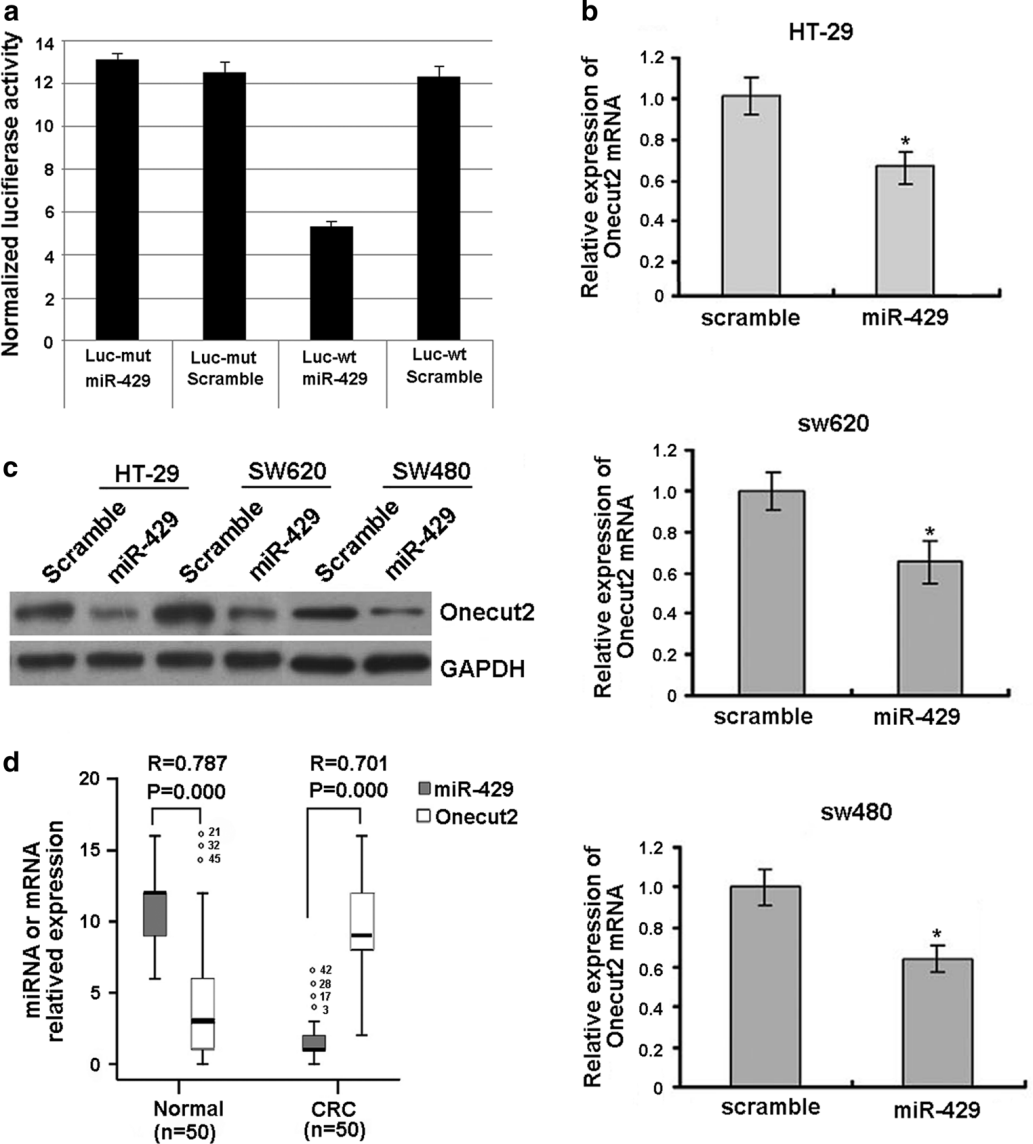

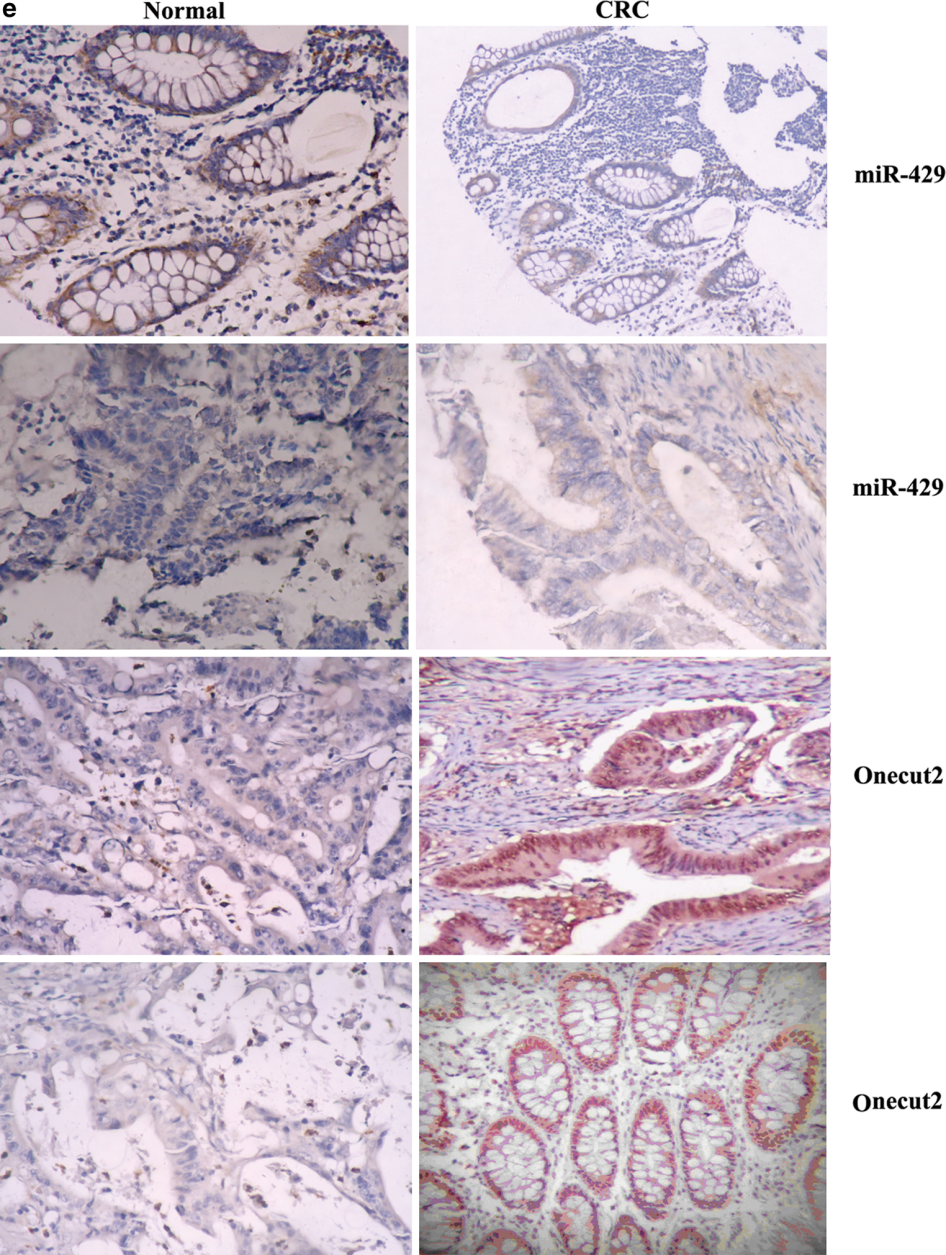
miR-429 targets transcript factor Onecut2 and is inversely correlated with the mRNA or protein levels of Onecut2 in CRC tissues. **a** Luciferase assay on SW620 cells, which were cotransfected with miR-429 and a luciferase reporter containing the full length of Onecut2 3′-UTR (Luc-wt) or a mutant (Luc-mut) in which the four nucleotides of the miR-429-binding site were mutated. Luciferase activities were measured 48-h post transfection. miR-429 markedly suppressed luciferase activity in Luc-wt reporter constructs. The data are mean ± SD for separate transfections (*n* = 6). **b** Onecut2 mRNA level was analyzed upon miR-429 or scramble transfection in HT-29, SW620, and SW480 cells by real-time PCR. All data are shown as mean ± SD. **P < 0.01. **c** miR-429 transfection affects Onecut2 protein levels by Western blot analysis. HT-29, SW620, and SW480 cells were transfected with miR-429 or scramble control. **d** Representative expression levels of miR429 and Onecut2 mRNA by real-time PCR in clinical specimens (*n* = 50), and the correlation between miR-429 and Onecut2 was analyzed by Spearman’s correlation test. **e** ISH detects the position and expression of miR-429, and IHC detects the position and expression of Onecut2 in CRC (103 cases) relative to paracancerous normal tissues (103 cases). *Scale bars* 100 μm

ONECUT2 is a member of the ONECUT transcription factor family, which was showed to be the core of the microRNA–gene networks by our previous study [24] and we proposed that ONECUT2 is one of the crucial targets of miR-429. To demonstrate this in CRC cells, the ONECUT2 complementary sequence or the mutant with a deletion of 4 nucleotides for the predicted binding of the miR-429 were cloned downstream of the firefly luciferase gene. SW620 cells were cotransfected with firefly luciferase constructs containing the ONECUT2 wild-type or mutated 3′UTRs and miR-429 or scramble oligonucleotides for 24 h, respectively. Luciferase activities were measured, as shown in Fig. 2a; significantly reduced luciferase activity was detected in cell transfected with wild-type ONECUT2 and miR-429, compared with the mutant sequence, indicating that the ONECUT2 complementary sequence contained the binding site for miR-429. The repressive effect of miR-429 on ONECUT2 expression was also measured in CRC cell lines HT-29, SW620, and SW480 by both real-time PCR and Western blot. As shown in Fig. 2b, c, miR-429 significantly reduced the expression of ONECUT2 in both transcriptional and protein level, respectively, which confirmed our previous bioinformatics prediction of the repressive effect of miR-429 on ONECUT2 expression.

We further confirmed that there was an inverse correlation between miR-429 levels and Onecut2 mRNA levels by real-time PCR in 50 CRC and 50 paracancerous normal tissue specimens (Fig. 2d). At the same time, we also used the CRC tissue array [24] to detect the expression of miR-429 by ISH and Onecut2 by IHC staining, and the results showed that miR-429 existed in cytoplasm and its expression was significantly decreased in CRC relative to paracancerous normal tissues; Onecut2 existed in nucleus and its expression was significantly increased in CRC relative to paracancerous normal tissues (Fig. 2e).

The above data suggest that downregulation of miR-429 contributes to CRC carcinogenesis by targeting regulating transcript factor Onecut2.

### Effect of miR-429 on cells mobility and invasion by target Onecut2 in CRC cells

**Fig. 3:**
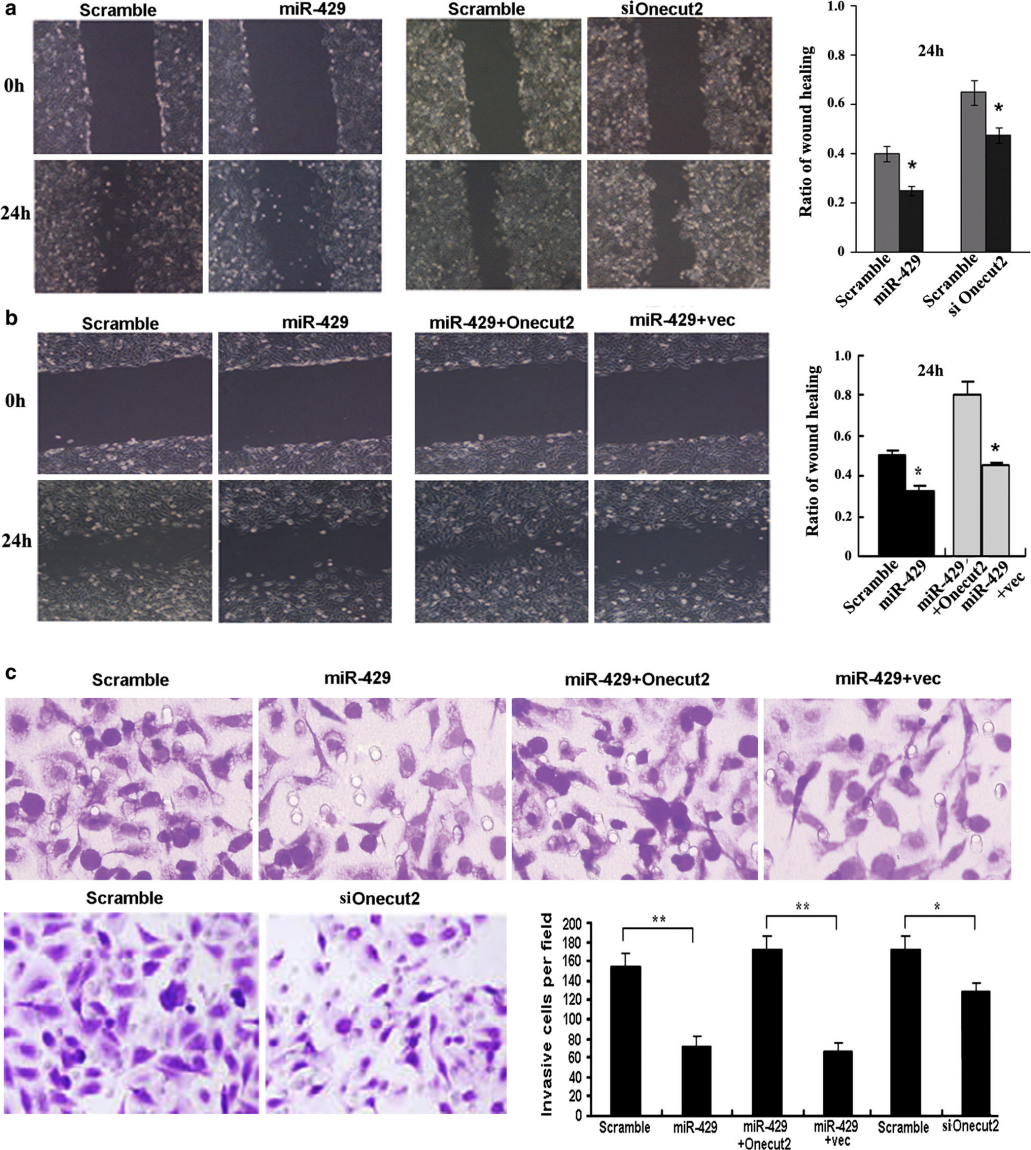
Effect of miR-429 on cells mobility and invasion by target Onecut2 in CRC cells. **a** Wound-healing assay analyzes the effect of miR-429 or si-Onecut2 on SW620 cells migration. *P < 0.05. **b** Wound-healing assay analyzes the rescued effect of Onecut2 overexpression on inhibitory healing induced by miR-429. *P < 0.05. **c** Matrigel invasion assay analyzes the rescued effect of Onecut2 overexpression on invasion inhibition induced by miR-429. **P < 0.01

The previous research indicated that although deregulation of miR-429 was not correlated with the lymphatic metastasis [24], miR-429 may be involved in the initiation of invasion of CRC. In light of the above findings, we decided to explore the biological significance of miR-429 in CRC tumorigenesis. Wound-healing assay was used to detect the impact of miR-429 on cell motility. SW620 cells were transfected with miR-429 mimics and the scramble control mimics, the wound healing was observed in an inverted microscope after scratching 0 and 24 h. After scratching 24 h, we showed that the wound healing extent with miR-429 mimics treatment significantly slows down that of the scramble treatment in SW620 cells (Fig. 3a). To analyze if miR-429 affected the wound healing through inhibiting Onecut2, we synthesized Onecut2 siRNA and constructed the pcDNA3.1–Onecut2 expression vector. SW620 cells were transfected with Onecut2 siRNA and scramble siRNA, and found that Onecut2 siRNA inhibited the wound healing (Fig. 3a); however, re-expression of Onecut2 rescued inhibitory effect of miR-429 on cell migration (Fig. 3b).

Next, we further to detect the effect of miR-429 on cell invasion by transwell matrigel invasion assay. Overexpression of miR-429 or siOnecut2 inhibited the in vitro invasion potential of SW620 cells, whereas re-expression of Onecut2 rescued this inhibition (Fig. 3c). The above data suggested that miR-429 can inhibit the motility and invasion of CRC cells by targeting Onecut2.

### MiR-429 inhibited the EMT induced by TGF-beta

**Fig. 4:**
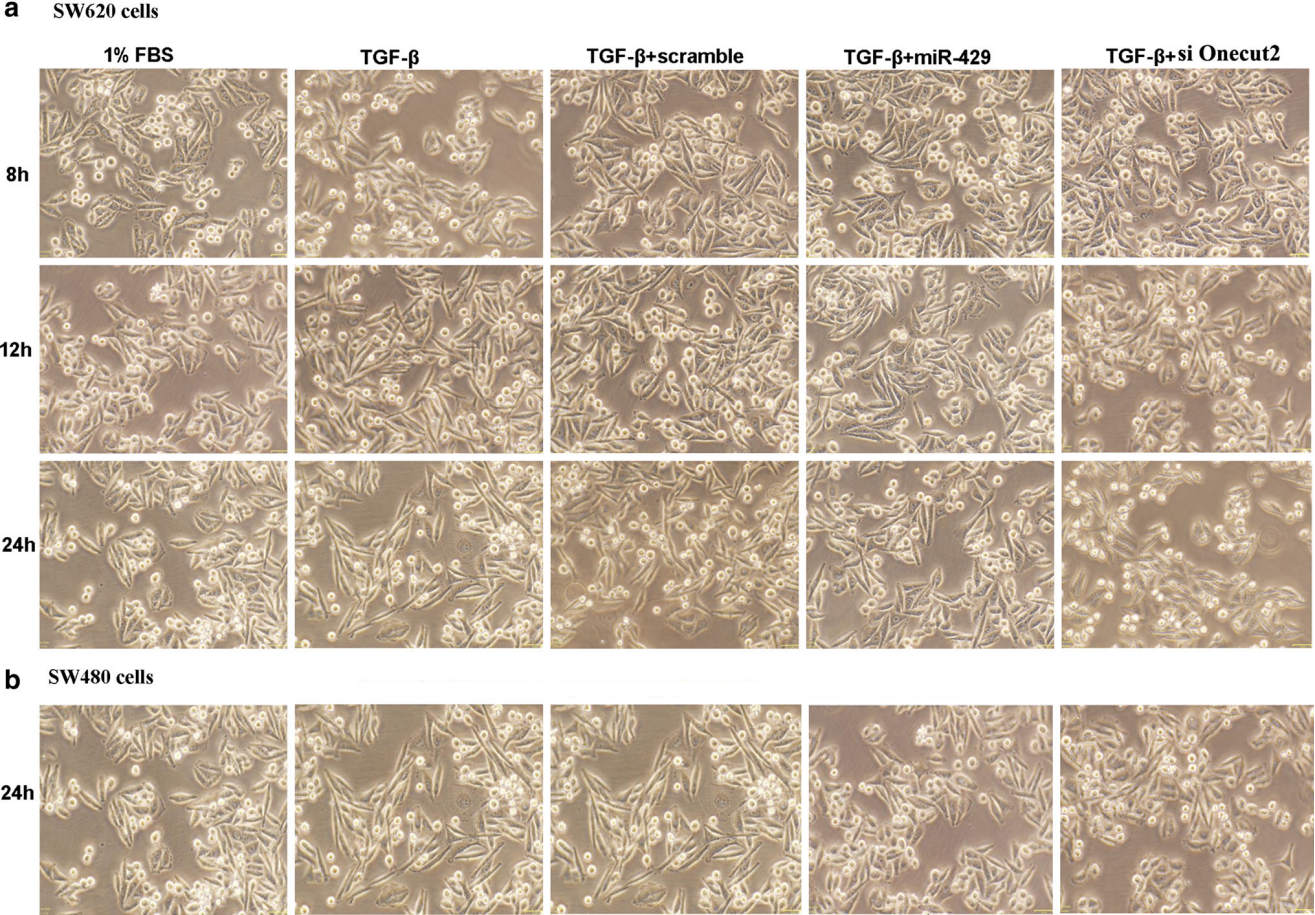
The morphology observation that miR-429 or siOnecut2 inhibits EMT induced by TGF-β in SW620 cells (**a**) and SW480 cells (**b**)

For EMT the most prominent hallmarks are loss of cell polarity, loss of cell–cell adhesion, and enhanced migration potential, eventually leading to increased motility and invasion of cancer cells [1]. The TGF-β is a pleiotropic cytokine, which drives the EMT mainly by repressing E-cadherin transcription and disrupting its localization at the plasma membrane, which influences adherens junctions [19]. The above data suggest that miR-429 can inhibit the motility and invasion of CRC cells by targeting Onecut2. Thus, in this section, we will focus on if miR-429 affects the TGF-β-induced EMT at different time points. The results presented in Fig. 4a and b demonstrate that TGF-β significantly resulted in a cells morphological phenotype of EMT after 24 h when SW620 or SW480 was treated with TGF-β, respectively; miR-429 mimics or siOnetcut2 inhibited SW620 or SW480 cells to undergo TGF-β-induced morphological change from a more polygoned, epithelial-like phenotype to an elongated, spindle-shaped, mesenchymal phenotype. In contrast, no change in morphology was detected in SW620 or SW480 cells transfected with the scramble control sequence.

### miR-429 regulated the expression of EMT-related transcript factors and genes in CRC cells

**Fig. 5:**
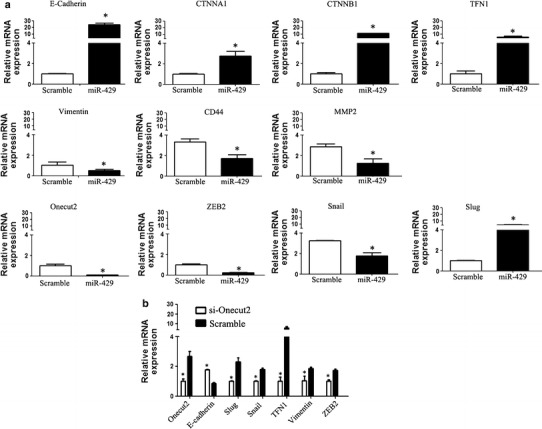
miR-429 regulates the expression of EMT-related marker genes by targeting Onecut2 in CRC cells. **a** miR-429 rescued the expression alteration of EMT-related marker genes induced by TGF-β1 in SW620 cells. *P < 0.05. **b** si-Onecut2 rescued the expression alteration of EMT-related marker genes induced by TGF-β1 in SW620 cells. *P < 0.05

EMT is characterized by loss of functional expression of E-cadherin and other epithelial markers such as cytokeratins or ZO-1; and induction of mesenchymal markers, notably vimentin, N-cadherin, α-smooth muscle actin, etc. [2]. EMT also involved in other adhesion and cytoskeletal-related genes such as CD44, MMP2, CTNA1, CTNB1, ZEB1, ZEB2, Snail, Slug, etc. Thus, we studied in depth the mechanism of miR-429 inhibiting EMT by targeting Onecut2. The data indicated that transforming growth factor TGF-β1 induced the change of the above EMT-related genes; however, miR-429 or si-Onecut2 rescued the genes expression alteration induced by TGF-β1 in SW620 cells. Both overexpression of miR-429 and interfering Onecut2 expression increased the expression of catenin molecular (E-cadherin, CTNNA1, and CTNNB1) and fibronectin (FN), and it also decreased the expression of heterogeneity adhesion molecule CD44, matrix metalloproteinase MMP2, Vimentin, and EMT-related transcript factors Slug, Snail, ZEB2, and Onecut2 (Fig. 5a, b).

## Discussion

miR-429 is a member of the miR-200 family, and positioned on chromosome 1. miR-429 is downregulated in renal cell carcinoma [3], nasopharyngeal carcinoma [4], Ehrlich ascites tumor cells [5], gastric cancer [3–6], and endometrial endometrioid carcinomas [7]. miR-429 and miR-200c are expressed in the luminal and basal type of breast cancer in contrast to malignant myoepithelioma, which revealed significantly lower levels potentially contributing to its mesenchymal phenotype [9].

In this study, we showed that miR-429 was significantly downregulated in II and III stages CRC cancer tissues when compared with adjacent normal tissue. We also used nude mouse xenograft models to confirm that miR-429 could suppress CRC cells tumorigenicity in vivo, suggesting that miR-429 could play a role in CRC tumorgenesis.

We next explored the possible targets of miR-429 in CRC cells through different computational algorithms. Taking into account our previous data that: Onecut2 existed in the core of the gene networks in colorectal carcinoma [24] and miR-429 was as the core of miRNA–RNA network of CRC network [24]; therefore, in-silico analysis revealed Onecut2 as a candidate target of miR-429, which attracted our attention immediately. We observed a substantial upregulation of Onecut2 protein in CRC tissues, and Onecut2 protein levels were inversely correlated with miR-429 levels. Importantly, we showed that miR-429 negatively regulated Onecut2 expression in mRNA and protein level in CRC cells, suggesting a role for miR-429 dysregulation in the pathogenesis of CRC.

The above data suggest that downregulation of miR-429 contribute to CRC carcinogenesis by targeting Onecut2. Our previous data also indicated that deregulation of miR-429 was not correlated with the lymphatic metastasis [24]; thus, we speculated that miR-429 may be involved in the initiation of EMT of CRC by targeting Oncut2. EMT is involved in both physiological and pathophysiological processes, whose most prominent hallmarks are loss of cell polarity, loss of cell–cell adhesion, and enhanced migration potential, eventually leading to increased motility and invasiveness of cancer cells [1]. We further to study the effect of miR-429 on cell migration, invasion, and EMT in CRC cells. Our data suggested that miR-429 inhibited the cells migration and invasion and reversed TGF-β-induced EMT changes in CRC cells. At the same time, interfering the endogenous Onecut2 also could inhibit the SW620 cell migration; overexpressed Onecut2 reversed the miR-429-mediated cell migration and invasion suppression.

TGF-β is a pleiotropic cytokine, which not only inhibits the cell proliferation, but also promotes the local invasiveness through induction of EMT or angiogenesis [2, 17, 18]. TGF-β drives the EMT by influencing adherens junctions either directly by interaction of TGF-β receptors and protein Par6, or by repressing E-cadherin transcription [19]. Component of an E-cadherin/catenin adhesion complex composed of at least E-cadherin/CDH1, β-catenin/CTNNB1, or γ-catenin/JUP, and potentially α-catenin/CTNNA1; the complex is located to adherens junctions [27]. TFN1, CD44, and MMP2 are all EMT markers genes, whose aberrant expressions were involved in the EMT of cancer [28–30]. Our data suggested that miR-429 overexpression and Onecut2 siRNA increased the expression of catenin molecular (E-cadherin, CTNNA1, and CTNNB1) and fibronectin (FN), and it also decreased the expression of heterogeneity adhesion molecule CD44 and matrix metalloproteinase MMP2. Although CTNNB1 gene that codes for β-catenin can function as an oncogene, its mutations is a cause which leads to growth of many tumors and especially CRC [31]; however, our data indicated that miR-429 increased the expression of CNNB1, one possible reason is that CNNB1 may be not mutated in SW620 cells.

The best characterized EMT inducers are zinc-finger transcription factors SNAI1/Snail and SNAI2/Slug, ZEB1/dEF-1, and ZEB2/SIP1, acting as repressors of E-cadherin transcription through interaction with conserved E-boxes in the E-cadherin promoter [3–5]. The previous studies showed that TGF-β1 induces the expression of ZEB1, ZEB2, and SLUG, but not SNAI in benign prostatic hyperplasia epithelial cell line BPH-1 Cells [32]. Our studies indicated that TGF-β1 could induce ZEB1, ZEB2, SNAI, and SLUG, miR-429 inhibited the expression of Onecut2, ZEB1, ZEB2, SNAI, and SLUG; whereas interfering the expression of Onecut2 had the consistent effects on the above transcript factors with the miR-429 overexpression.

Taken together, our data showed that transcript factor Onecut2 is involved in the EMT, migration, and invasion of CRC cells, miR-429 inhibits the initiation of EMT by targets Onecut2, and Onecut2-mediated EMT-related genes and transcript activators. In the next study, we will demonstrated the potential mechanism by which miR-429 is downregulated in colorectal carcinoma, the downregulated expression of miR-429 may be correlated with the aberrant regulation of transcript factor or long non-coding RNAs.

## Electronic supplementary material

Below is the link to the electronic supplementary material.

Supplementary material 1 (DOC 4668 kb)
