# Correction to: miR-429 inhibits cells growth and invasion and regulates EMT-related marker genes by targeting Onecut2 in colorectal carcinoma

**DOI:** 10.1007/s11010-021-04185-3

**Published:** 2021-06-10

**Authors:** Yingnan Sun, Shourong Shen, Xiaoping Liu, Hailin Tang, Zeyou Wang, Zhibin Yu, Xiayu Li, Minghua Wu

**Affiliations:** 1 Hunan Key Laboratory of Nonresolving Inflammation and Cancer, Changsha, Hunan People’s Republic of China; 2 Department of Gastroenterology, Third Xiangya Hospital, Central South University, Changsha, 410013 Hunan People’s Republic of China; 3 Disease Genome Research CenterKey Laboratory of Carcinogenesis and Cancer Invasion, Ministry of EducationKey Laboratory of Carcinogenesis, Ministry of Health, Cancer Research Institute, Central South University, Changsha, Hunan People’s Republic of China; 4 Sun Yat-Sen University Cancer Center, State Key Laboratory of Oncology in South China, Collaborative Innovation Center for Cancer Medicine, Guangzhou, Guangdong People’s Republic of China

## Correction to: Molecular and Cellular Biochemistry (2014) 390:19–30 10.1007/s11010-013-1950-x

In the original publication of the article, Fig. 4 was published incorrectly. The correct version of Fig. 4 is provided in this correction.

**Fig. 4:**
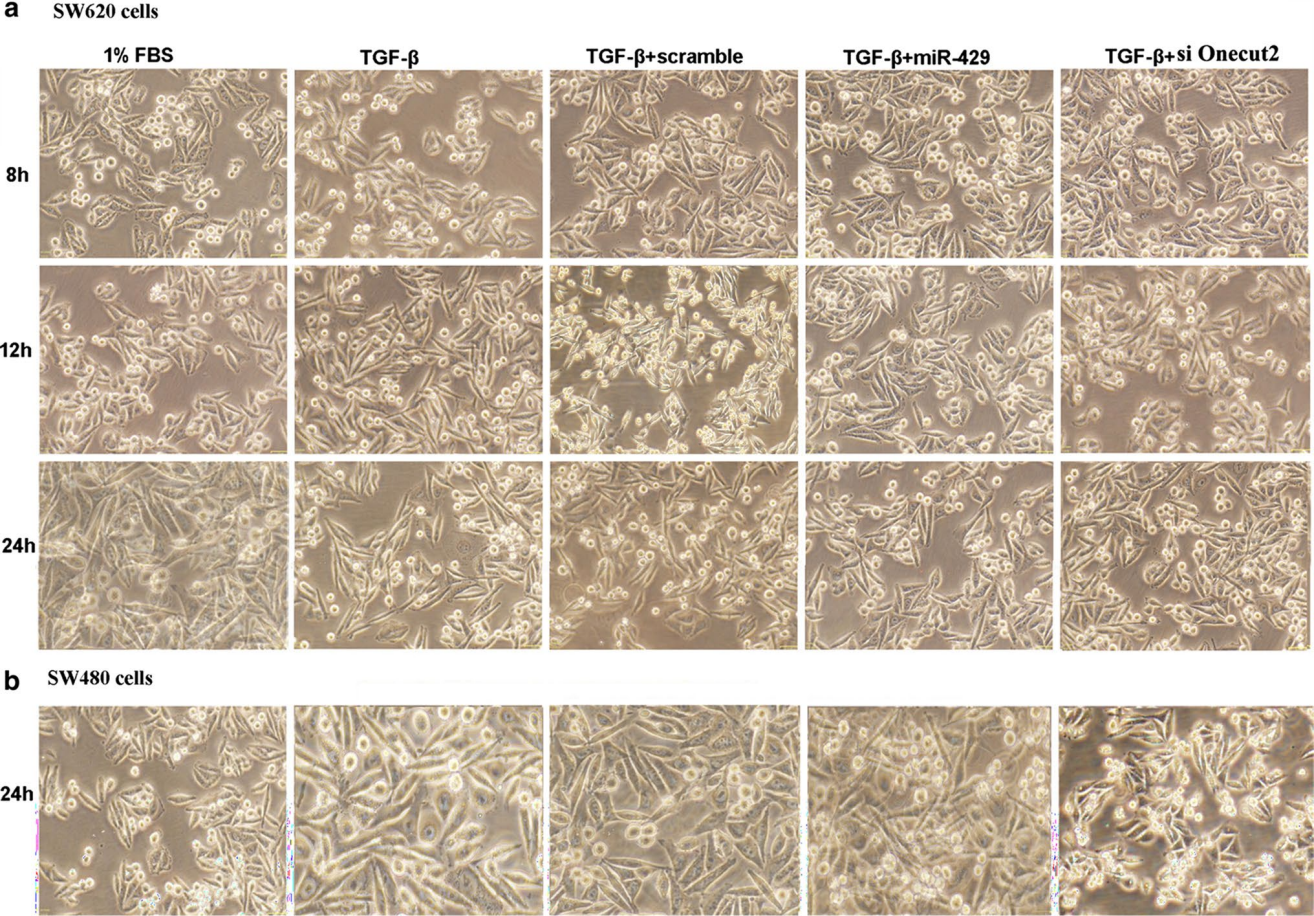
The morphology observation that miR-429 or siOnecut2 inhibits EMT induced by TGF-β in SW620 cells (**a**) and SW480 cells (**b**)

