# Supplementary material for: miR-429 inhibits cells growth and invasion and regulates EMT-related marker genes by targeting Onecut2 in colorectal carcinoma
Source: Mol Cell Biochem. 2014 Jan 10;390(1-2):19–30. doi: 10.1007/s11010-013-1950-x (PMC3972435; doi:10.1007/s11010-013-1950-x)
Supplement: Supplementary file 1 — Supplementary material 1 (DOC 4668 kb) [file 11010_2013_1950_MOESM1_ESM.doc]

**FigureS1**


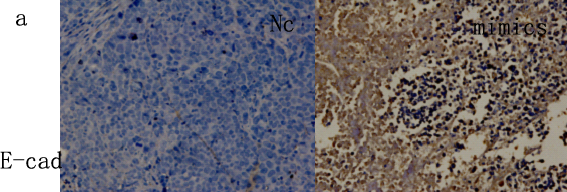


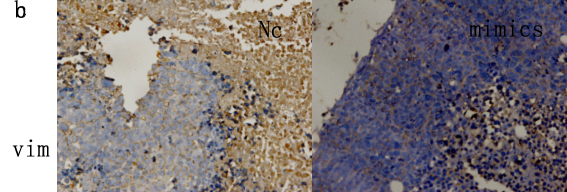


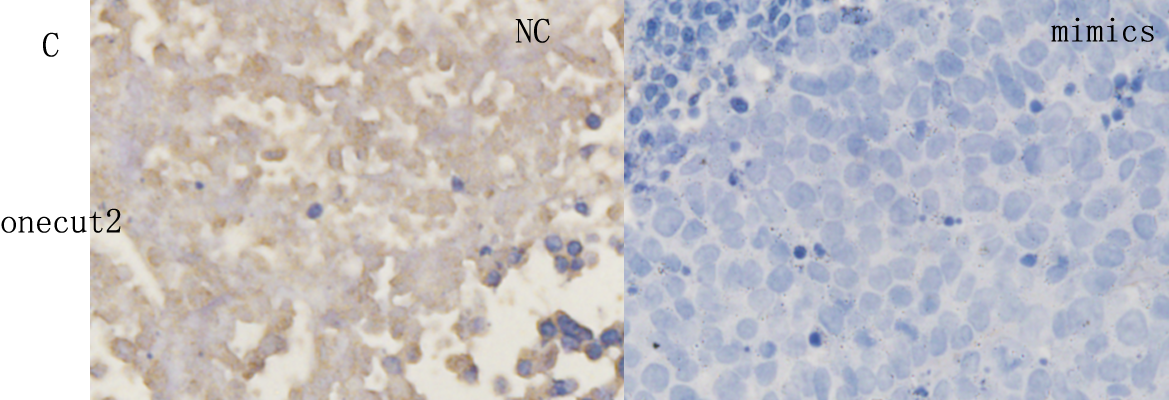


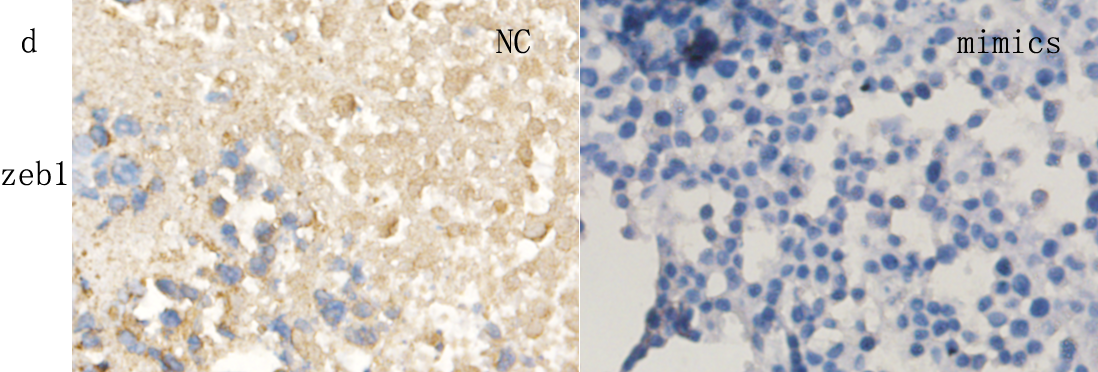

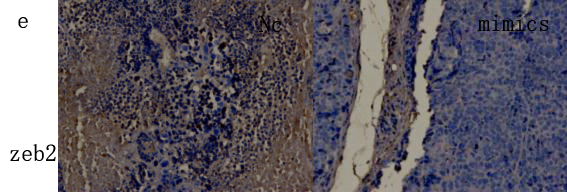


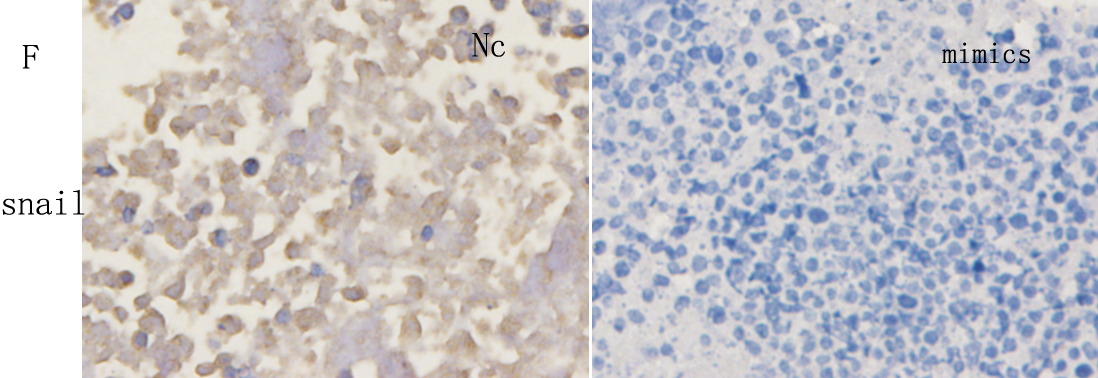


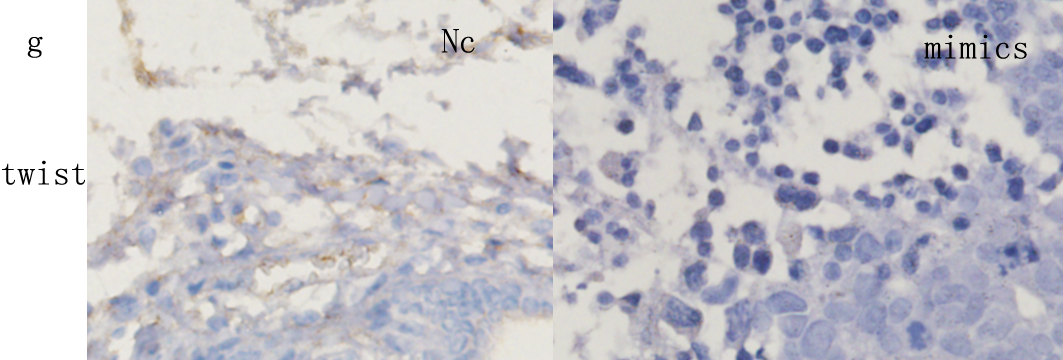


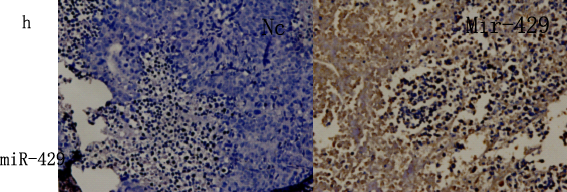


Figure S1 The effect of miR-429 on expression of EMT-related Marker genes in sw620 nude mice subcutaneous transplanted tumors with miR-429 treatment.

MiR-429 regulated the expression of E-cadherin,inhibited the expression of SIP1、VIM、SNAIL、ONECUT2、TWIST、 ZEB1 （Scale bar:200μm）

a, b, c, d, e, f and g were detected by IHC and h was detected by ISH.

**Figure S2**


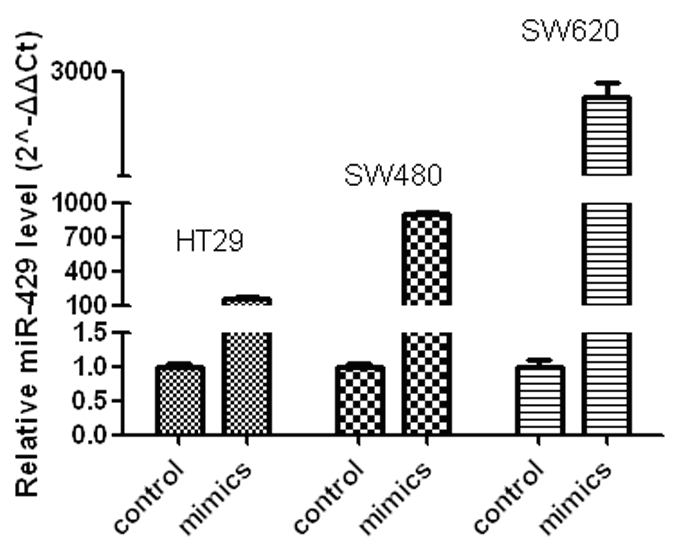


Figure S2: miR-429 was tranfected into the colorectal carcinoma cells (SW480, SW620 and HT-29), respectively, real-time PCR detect the expression of miR-429.
